# Editorial: Medicinal Plants and Marine-Derived Natural Products as Cancer Chemopreventive Agents

**DOI:** 10.3389/fphar.2022.900275

**Published:** 2022-06-02

**Authors:** Riaz Ullah, Najeeb Ur Rehman, Fatemeh Jamshidi-Adegani, Ahmed Bari

**Affiliations:** ^1^ Medicinal Aromatic and Poisonous Plants Research Center (MAPPRC), College of Pharmacy, King Saud University, Riyadh, Saudi Arabia; ^2^ Department of Pharmacognosy, College of Pharmacy, King Saud University, Riyadh, Saudi Arabia; ^3^ Natural Products Laboratory, Natural and Medical Sciences Research Center, University of Nizwa, Nizwa, Oman; ^4^ Laboratory for Stem Cell and Regenerative Medicine, Natural and Medical Sciences Research Center, University of Nizwa, Nizwa, Oman; ^5^ Department of Pharmaceutical Chemistry, College of Pharmacy, King Saud University Riyadh, Riyadh, Saudi Arabia

**Keywords:** medicinal plant, natural product, marine life, anticancer, chemopreventive

Cancer, irrespective of the advance technologies, is the second most common leading source of death and mortality globally and growing with changing nutrition, lifestyle, and global warming. Indeed, the preclusion of cancer is extremely superior to treatment. Many cancer patient deaths result from resistance acquired during treatments. For cancer treatment, early stage diagnosis and conclusive tumor eradication by radiation therapy/surgical resection built greatest hope. Cancer chemoprevention is the use of natural or synthetic compounds to suppress, prevent, or reverse the process of carcinogenesis and is an alternative significant approach for simplifying this formidable public health burden (Shankar et al., 2012). The phytoconstituents that retard the instigation point are commonly known as preventive agents, and the ones distressing the raise phase are known as suppressing agents (Chen and Kong, 2005). The awareness of chemo prevention is getting more fame due to its achievement in reducing the occurrence of diseases.

Recently, natural products (NPs) acquired an essential stage in cancer treatment due to multi-targeting efficacy, ease in availability, high potential of chemo-sensitization, cost-effectiveness, no toxicity, and drug resistance. Recently, numerous studies have determined the importance of natural products and their bioactivities against chronic metabolic diseases, and some prominent bioactive compounds in herbal medicines can reduce the effect of such diseases. However, their use is limited because of their low solubility and limited half-life (Shankar et al., 2012). Based on these aspects, new curative therapeutic strategies are immediately required that overcome the anticancer therapy resistance and have longer efficacy, lesser toxicity as well as improved selectivity.

Since ancient times, NPs and their biosynthetic modifications have played a dominant role and key contribution to pharmacotherapy, in particular for cancer and infectious diseases. NPs have built up significant and substantial improvement in the management of cancer therapy. They are considered a significant basis of novel prime phytoconstituents for chemo preventive medications (Cragg and Pezzuto, 2016). According to one report, over 60% of the existing drugs for cancer treatment were obtained in somehow from natural sources (Newman and Cragg, 2012), and around 80% of the world’s population rest on medicines obtained from plants for the health care system (Torre et al., 2015; Amrati et al., 2021). Hence, it is essential to grow innovative approaches for the improvement of cancer cure and NPs as a gifted source for effective development of drugs. NPs originated from plants and marine life have recently induced extensive attention as 70% of the earth is covered by water. This is representing that 95% of NPs derived from marine organisms have been explored with distinct pharmacological actions. With the increased awareness of herbal medicines roles in health and nutrition, scientists are dedicated to researching the functional or bioactive metabolites in medicinal plants.

The marine microorganisms, in addition to terrestrial plants, have yielded remarkable cancer chemotherapeutic agents (Yun et al., 2019). Over 60% of the current anticancer drugs were derived from natural sources. Nature continues to be a rich source of biologically active chemo types, while almost few of the original isolated NPs have been developed into clinically effective drugs. These unique molecules often serve as models for developing more successful analogues and prodrugs via chemical methodologies though total/combinatorial synthesis, or biosynthetic pathways. Some of the NPs including curcumin, resveratrol, tryptanthrin, kaempferol, gingerol, emodin, quercetin, and genistein are in preclinical stage as prospective chemo preventive agents (Shankar et al., 2012). Furthermore, enhancements/improvements in formulation might cause more effective and selective administration of the drug to patients, which can lead to the progress of efficacious targeted therapies. The crucial role played by the NPs isolated from the natural sources in the optimization of novel molecular leads has been broadly reviewed (Steward and Brown, 2013).

Aim of this special issue was to collect data on active NPs or extracts from natural sources, including marine and plants as cancer chemo preventive agent. In this special issue, four papers have been published which were evaluated by expert reviewers. Xi et al. reported Ciji-Huaai-Baosheng II Formula (CHB-II-F) as a traditional Chinese medicine particularly targets certain aspects of chemotherapy-induced effects in cancer patients. The obtained result displayed that CHB-II-F strengthens the inhibitory effect of 5-FU on tumor (caused by chemotherapy), by enhancing the pathological injury of gastrointestinal tract, as well as controlling and regulating the secretion of gastrointestinal hormones. It may simplify the chemotherapy-induced anorexia through peripheral and appetite regulatory factors in the feeding region of hypothalamus central nervous system (Xi et al.). Shi et al. demonstrated that Gallic Acid (GA, a natural phenolic acid) suppressed tumorigenesis (*in vitro* and *in vivo*) through lncRNA MALAT1-Wnt/β-catenin axis in HCC. This study provides an original perception about cancer prevention and to develop a novel synergistic intervention between GA and traditional chemotherapy agents (Shi et al.).

Yang et al. studied the inhibitory effect (*in vitro* and *in vivo*) of the astragalin (flavonoid) on proliferation and movement of human colon cancer (HCT116) cells. The results have shown that astragalin expressively protect the proliferation and diffusion of HCT116 cells by convincing apoptosis and cell cycle through modulation of protein expression. Therefore, astragalin might be considered as a promising plant-derived antitumor drug for colon cancer.

Tao et al. studied that the ethyl acetate extract of *Celastrus orbiculatus Thunb* can significantly persuade apoptosis of human gastric cancer cell (*in vivo* and *in vitro*) via inhibiting BPH expression. The results determined that the PHB intimation in gastric cancer selected samples was significantly higher than the corresponding adjacent tissues, the AGS cell proliferation was significantly inhibited. Furthermore, the *in vivo* experiments attributed that the progression of subcutaneous transplanted tumor was noteworthy inhibited by the PHB knockdown and by the ethyl acetate intragastric administration.

In conclusion, cancer is still a major clinical problem worldwide. The different articles published in this special issue determined that still researchers around the world are working on the interaction of NPs with different cancer cell lines (*in-vivo* and *in-vitro*) for effective cancer treatment in order to get patient compliance. In addition to that, we can also suggest that the combination of drugs, plant extracts and marine species with other techniques could provide better results in these conditions.
